# Room-Temperature Magnetism of Ceria Nanocubes by Inductively Transferring Electrons to Ce Atoms from Nearby Oxygen Vacancy

**DOI:** 10.1007/s40820-015-0056-2

**Published:** 2015-08-19

**Authors:** Yue Kang, Qiang Leng, Donglin Guo, Dezhi Yang, Yanping Pu, Chenguo Hu

**Affiliations:** 1 Department of Applied Physics, Chongqing University, Chongqing, 400044 People’s Republic of China; 2 School of Public Affairs, Chongqing University, Chongqing, 400044 People’s Republic of China

**Keywords:** UV irradiation, Oxygen vacancies, Saturation magnetism, Spin direction, Superexchange interaction

## Abstract

Ceria (CeO_2_) nanocubes were synthesized by a hydrothermal method and weak ferromagnetism was observed in room temperature. After ultraviolet irradiation, the saturation magnetization was significantly enhanced from ~3.18 × 10^−3^ to ~1.89 × 10^−2^ emu g^−1^. This is due to the increase of oxygen vacancies in CeO_2_ structure which was confirmed by X-ray photoelectron spectra. The first-principle calculation with Vienna ab-initio simulation package was used to illustrate the enhanced ferromagnetism mechanism after calculating the density of states (DOSs) and partial density of states (PDOSs) of CeO_2_ without and with different oxygen vacancies. It was found that the increase of oxygen vacancies will enlarge the PDOSs of Ce 4f orbital and DOSs. Two electrons in one oxygen vacancy are respectively excited to 4f orbital of two Ce atoms neighboring the vacancy, making these electron spin directions on 4f orbitals of these two Ce atoms parallel. This superexchange interaction leads to the formation of ferromagnetism in CeO_2_ at room temperature. Our work indicates that ultraviolet irradiation is an effective method to enhance the magnetism of CeO_2_ nanocube, and the first-principle calculation can understand well the enhanced magnetism.

## Introduction

Nanocrystalline ceria (CeO_2_) has attracted much attention due to its fascinating mechanical and physic-chemical properties, as well as wide applications in various fields, including polishing materials [1, 2], automobile exhaust catalysts [3–5], fuel cell materials [6, 7], gas sensors [8, 9], high temperature superconducting materials [10], ultraviolet ray detectors, etc. [11, 12]. Recently, Zhang et al. [13] reported that CeO_2_ nanoparticles have great potential for multifunctional therapeutic applications in cancer therapy. Flytzani-Stephanopoulos et al. [14] used different nanostructured CeO_2_ materials as a support to deposit gold clusters and found that Au/CeO_2_ catalysts showed a strong support shape effect in the water-gas shift reaction. Most of the properties of cerium oxide was found to be related with oxygen vacancies in the structure [15–18].

It is well known that CeO_2_ with perfect structure is paramagnetic. When one of oxygen atoms is removed, two electrons will be left and localized strongly at the f-level traps on two Ce atoms. This will cause the formal valence of two neighboring Ce atoms changing from +4 to +3, and therefore resulting in the formation of magnetism. Lee and co-workers [17] examined the localization behavior of CeO_2_ with various degrees of oxygen deficiency as well as the associated magnetic properties and their origins using first-principles methods in the Vienna ab-initio simulation package (VASP). Choudhury and Choudhury [10] investigated how oxygen vacancies and cationic Ce^3+^ defects in ceria affect the band gap property by changing its structural regularity and then control its visible luminescence. In addition, vacancies in some other non-magnetic materials can also induce magnetism. Si et al. [19] found that vacancies in hexagonal boron nitride nanosheets (h-BN) could generate magnetic moments and form ferromagnetism, and they conducted some experiments to test their discovery.

Although some methods could generate magnetism change such as spin-reorientation transition (SRT) in ultrathin Ni films grown on Cu (100) [20], few papers report the experimental methods of obtaining oxygen vacancies and changing the valence state. Recently, Qin et al. [21] improved the magnetic properties of semiconductors and found that UV irradiation could significantly enhance the magnetism of the (111) twinned BaTiO_3_ crystallites. They demonstrated that magnetism might originate from the increase in oxygen vacancies and Ti^3+^ cations formed by capturing an excited electron from Ti^4+^ under UV irradiation. Enlightened by this report, we speculate that the valence state might also change (from Ce^4+^ to Ce^3+^) under UV irradiation. Herein, the optical and magnetic properties of CeO_2_ under UV irradiation were investigated and the electron density of states (DOSs) and the electron partial density of states (PDOSs) were calculated via density functional theory for a better understanding of the phenomenon.

## Experimental Details

### Synthesis of CeO_2_ Nanocubes

The uniform organic-capping-free CeO_2_ nanocubes were synthesized using a hydrothermal process [22]. Briefly, 434.3 mg of Ce(NO_3_)_3_·6H_2_O and 1.6 mg of Na_3_PO_4_ were mixed in 40 mL deionized water and sonicated for 30 min. The mixed solution was then transferred into a Teflon-lined autoclave with 50 mL capacity. The vessel was sealed and heated at 200 °C for 24 h in an electric furnace. Then the product was collected and cleansed by centrifugation at 10,000 r min^−1^ for 5 min and re-dispersed twice in ethanol. The precipitate was then calcined at 400 °C for 4 h and collected for further characterization.

### UV Irradiation on CeO_2_

The dried sample was uniformly distributed in a square crucible and put in a darkroom to irradiate for 24 h under the UV lamp.

### Characterization of CeO_2_

The powder X-ray diffraction (XRD) patterns were recorded on PANalytical Empyrean equipped with a PIXcel with a 1D detector at 40 kV and 40 mA. Field emission scanning electron microscopy (FESEM) was carried out with a Nova 400 Nano microscope to analyze the morphology and size of the product. Transmission electron microscopy (TEM) was used to analyze the morphology and size of the samples with JEOL-400EX. The X-ray photoelectron spectra (XPS) were conducted by an ESCALab MKI X-ray photoelectron spectrometer, using non-monochromatized Mg Kα X-ray as the excitation source. The reflection spectrum of the sample was measured using UV-3600 (SHIMADZU) UV–VIS–NIR Spectrophotometer. Magnetism measurement was carried out on superconducting quantum interference device (SQUIDs) measurement.

### Computation of Magnetism

First-principles methods, implemented in the Vienna ab-initio simulation package (VASP) [23], were used to study the magnetism of CeO_2_ without and with oxygen vacancies. Because of the strong Coulomb interaction of the localized Ce 4f electrons, the standard density functional theory (DFT) calculations with the correction of Hubbard *U* parameter (DFT+*U*) were employed [24, 25]. We chose *U* = 7 eV [26] to improve the prediction of the computation. And a 500 eV plane-wave energy cutoff was used to expand the electronic wave functions. The 7 × 7 × 7 Monkhorst–Pack grid was used for the sampling of the Brillouin zone during geometrical optimization. A supercell of 2 × 2 × 2 without any vacancy and with different oxygen vacancies was built to simulate the magnetic properties of CeO_2_. All the atomic positions and lattice parameters were relaxed and optimized until the convergence of force reaches 0.01 eV Å^−1^.

## Results and Discussion

**Fig. 1:**
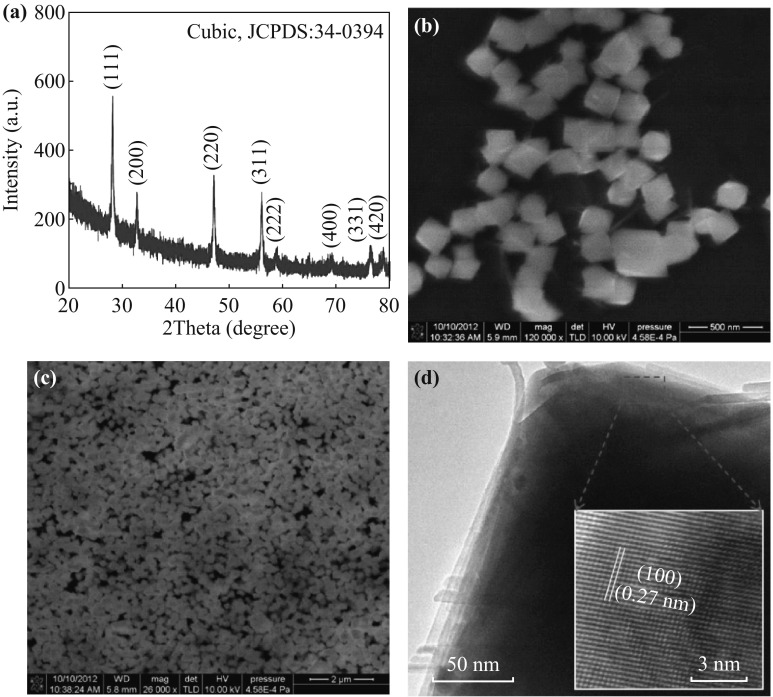
**a** XRD, **b**, **c** FESEM images, and **d** TEM image of the CeO_2_ sample

A typical XRD pattern of the sample is shown in Fig. 1a. All the peaks can be indexed to the cubic phase (Fm3m, JCPDS 34-0394) of CeO_2_ without any impurity peaks. FESEM images of the synthesized CeO_2_ sample, as shown in Fig. 1b, c, demonstrate that the particles are quite uniform and have regular cube-like morphology with a size of 200 nm. TEM image in Fig. 1d also shows clear cubic shape of CeO_2_ particle. The inset is the high-resolution TEM image of the selected area, which has lattice planes (100) with *d*-spacing of 0.27 nm.

**Fig. 2:**
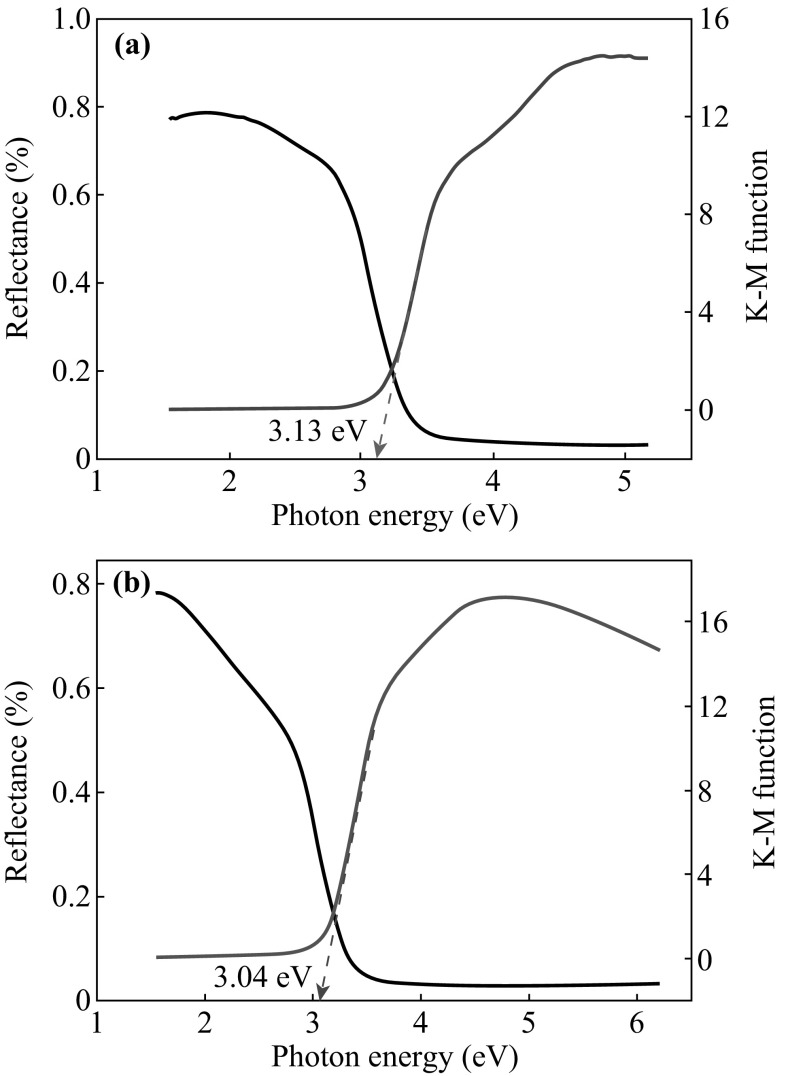
UV–Visible reflectance spectrum and Kubelka–Munk function of the CeO_2_ sample before (**a**) and after (**b**) UV irradiation

Figure 2a, b show UV–Vis reflectance spectra and Kubelka–Munk functions of the CeO_2_ nanocubes without and with UV irradiation. Extrapolating the linear part of Kubelka–Munk function, which is actually the ratio of the absorption and scattering factors from the optical diffuse reflectance spectrum [27], the energy gap of the CeO_2_ can be evaluated as 3.13 and 3.04 eV for the CeO_2_ sample without and with UV irradiation, respectively (Fig. 2a, b) [28, 29]. The reduced band gap can be attributed to the defect energy level, as some previous studies have reported that oxygen deficiencies create additional energy level within the forbidden energy gap [30, 31].

**Fig. 3:**
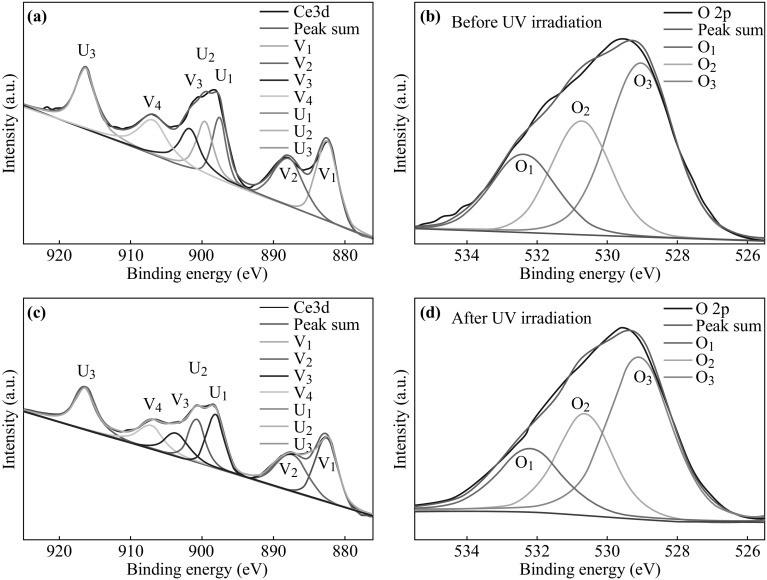
**a** Ce 3d core level, and **b** O 2p core level for the CeO_2_ sample before UV irradiation. **c** Ce 3d core level, and **d** O 2p core level for the CeO_2_ sample after UV irradiation

Since the intensity of Ce 4p and Ce 4d is much lower compared to that of Ce 3d, we choose Ce 3d signal in XPS results to analyze further. Figure 3a shows the photoelectron spectrum of Ce 3d core level before UV irradiation, among which V and U are for Ce^3+^ and Ce^4+^ states, respectively. The binding energy peaks labeled as V1 and V2 are located at 883.26 eV and 902.81 eV, indicating the Ce^3+^3d_5/2_ and Ce^3+^3d_3/2_. The peaks (U1, U2 U3, U4, U5, and U6) of Ce^4+^3d_5/2_ and Ce^4+^3d_3/2_ are respectively shown at binding energy of 881.48, 888.34, 897.73, 899.85, 907.05, and 916.34 eV [32–34]. Compared with the sample after UV irradiation in Fig. 3b, it can be found that positions of Ce 3d peaks are nearly unchanged. However, the content of elements changes and the intensity ratio of Ce^3+^ to Ce^4+^ increases from 0.260 to 0.332. This may be ascribed to the increase of Ce^3+^ ions after UV irradiation. Figure 3c shows the O 2p core level of the sample before UV irradiation, labeled as O1, O2, and O3, which indicates the valence of O is -2. The levels of O 2p peaks at 528.83 and 530.86 eV are ascribed to O^2−^ ions which are related to Ce^4+^ and Ce^3+^ ions [33], while the peak of 532.47 eV is attributed to the oxygen absorbed onto the surface of the samples [35, 36]. Same with Ce 3d, the positions of O 2p peaks after UV irradiation do not shift while the relative intensity of these peaks changes (Fig. 3d), which indicates the change of element quantity. In calculation results, the intensity ratio of O2 to O1 increases from 1.183 to 1.291, demonstrating that the O^2−^ ions related to Ce^3+^ increase, which confirms the increase in oxygen vacancies. All the curve-fitting processes in Fig. 3 use a mixed Gaussian–Lorentzian simulation method with the same full width at half-maximum.

**Fig. 4:**
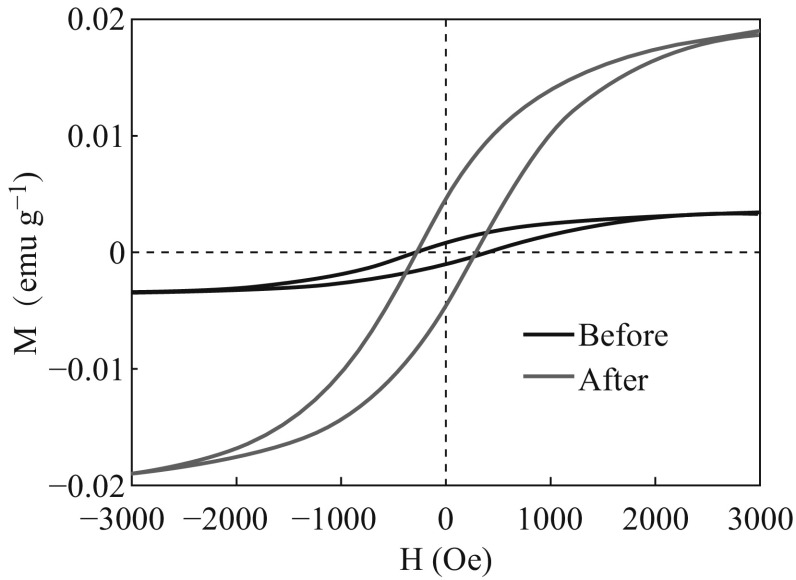
Hysteresis loops of the CeO_2_ sample before and after UV irradiation measured at room temperature

Figure 4 shows the hysteresis loops of sample from −15,000 Oe to +15,000 Oe before and after UV irradiation. The saturation magnetization of the sample before UV irradiation is 3.18 × 10^−3^ emu g^−1^, while it was greatly enhanced to 1.89 × 10^−2^ emu g^−1^ after UV irradiation. This may relate to the increase of oxygen vacancies caused by UV irradiation.

From the above discussion, we have demonstrated that UV irradiation can generate oxygen vacancies, and oxygen vacancies will induce the enhancement of magnetism. In order to understand the mechanism, electron DOS, projected density of states (PDOS), and the magnetism of the structure were calculated using the first-principle calculation in the VASP. In all calculations, the projector augmented wave method (PAW) [37] with the frozen-core approximation was used for the ion–electron interactions. Exchange correlation interactions were described by the generalized gradient approximation (GGA) [38].

**Fig. 5:**
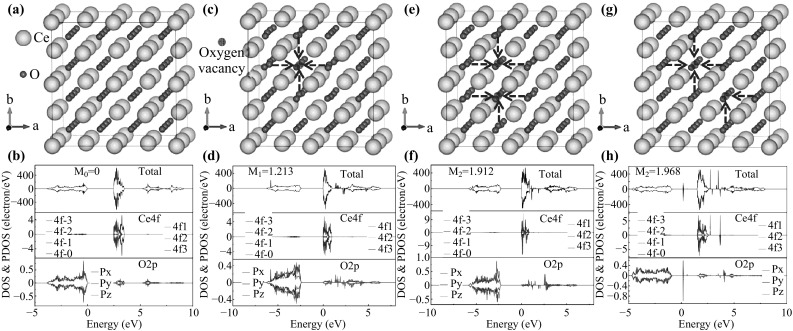
The schematic of CeO_2_ supercell without any oxygen vacancy (**a**), with one oxygen vacancy (**c**), two oxygen vacancies (**e**, **g**), and their corresponding DOSs and PDOSs (**b**, **d**, **f**, and **h**)

The DOSs and PDOSs of CeO_2_ without and with one oxygen vacancy and two oxygen vacancies were calculated and the results are shown in Fig. 5. One can see the perfect structure of tetrahedral CeO_2_ supercell with 32 cerium atoms and 64 oxygen atoms. Each oxygen atom is surrounded by four cerium atoms with the same distance. To simplify the atom orbitals, the f orbitals of Ce 4f are marked as 4f-3 ~ 4f3 and the p orbitals of O 2p are marked as P_x_ ~ P_z_ as shown in Fig. 5b. From the total DOS of CeO_2_, all the electron spin-up and spin-down states are symmetric and show no magnetism in the perfect CeO_2_ cell. Comparing the Ce 4f state with O 2p, we have observed that O 2p contributes more to the total DOS at the top valence band than the Ce 4f. While at the bottom conduction band the majority was formed by Ce 4f. The integrative action of Ce 4f and O 2p makes the CeO_2_ non-magnetic. When one oxygen is removed, an oxygen vacancy is generated, as shown in Fig. 5c. Several PDOSs of Ce 4f are symmetric except f-3, f-2, and f3, which generate most of the magnetism of CeO_2_ with one vacancy. From the PDOSs of O 2p, the P_x_, P_y_, and P_z_ states are symmetric at the top valence band constituting most of the valence band of CeO_2_, as can be seen from Fig. 5d. Meanwhile, at the bottom conduction band, O 2p exhibits slight asymmetry which contributes to the formation of the conduction band of CeO_2_. With one oxygen vacancy, the calculation of magnetism of the sample is about 1.213 μB. After another oxygen atom is removed, two oxygen vacancies are formed. Herein, we choose two cases to analyze the magnetism as depicted in Fig. 5e, g. Comparing the DOSs and PDOSs of Ce 4f and O 2p in Fig. 5f, h, it is not difficult for us to discover that all their electron spin-up and spin-down in f orbitals of Ce 4f are asymmetric, generating most of the magnetism of CeO_2_. Same as the situation in Fig. 5b, the electron spin-up and spin-down of O 2p are symmetric at the top valence band and reveal a bit of asymmetry at the bottom conduction band. Since the asymmetry of PDOSs in f orbitals in Fig. 5h is higher than that of PDOSs in f orbitals in Fig. 5f, the magnetizaiton of the sample in Fig. 5h is larger than that in Fig. 5f in accordance with the values in the figure (*M*_2_ = 1.912 μB, *M*_2′_ = 1.968 μB). Moreover, the magnetization of the sample with two oxygen vacancies is obviously larger than that with one oxygen vacancy, which well matches our demonstration that the enhancement of the magnetism of CeO_2_ after UV irradiation is due to the generation of oxygen vacancies. Since the oxygen vacancy exhibits positive electricity, it attracts nearest oxygen atoms. The blue arrows in Fig. 5c, e, g demonstrate the displacement of the neighboring oxygen atoms. All the structures of CeO_2_ without and with oxygen vacancies are optimized in the calculations of the DOSs, PDOSs, and magnetism.

**Fig. 6:**
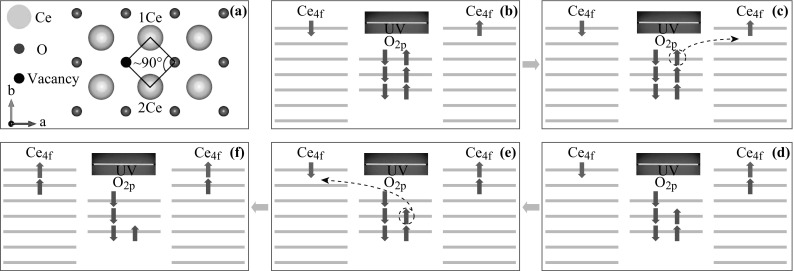
The schematic of superexchange interaction among 1Ce, 2Ce, and O (**a**), and the process of superexchange interaction of Ce–O–Ce (**b**–**f**)

Figure 6a shows the schematic of superexchange interaction among 1Ce, 2Ce, and O atoms. The two Ce atoms are respectively reduced by two electrons from the oxygen vacancy, forming a Ce–O–Ce bond together with the neighboring O atom, which favors a 90° superexchange interaction, leading to magnetism [17, 39]. Figure 6b–f display the process of superexchange interaction of Ce–O–Ce. First, one electron on one O 2p orbital is excited by UV irradiation to one 4f orbital of 1Ce atom (Fig. 6c), and its spin direction is parallel to the electron on Ce 4f orbital based on Hund’s rule [40] (Fig. 6d). Since the Ce–O–Ce bond angle is close to 90°, the electron with the same spin direction on another O 2p orbital is exchanged with 4f orbital of 2Ce atom (Fig. 6e), which makes the spin direction of the electron on 2Ce 4f orbital parallel to it (Fig. 6f). Therefore, the magnetic moments of these two Ce atoms have the same direction, generating ferromagnetic coupling. With the increase in oxygen vacancies, the tetrahedral structure of the sample is further destroyed, making the Ce atoms close to the oxygen vacancies, which increases the total magnetic momentum. Back to Fig. 5, as UV irradiation produces more oxygen vacancies in the structure of CeO_2_, the electron spin on Ce 4f orbitals becomes more complicated, making the PDOSs of Ce 4f more asymmetric. Thus UV irradiation can enhance the magnetism by producing oxygen vacancies.

## Conclusions

In this work, uniform CeO_2_ nanocubes were synthesized and oxygen vacancy can be increased and therefore the magnetism was enhanced by UV irradiation. The first-principle calculation was used to explain the magnetism enhancement mechanism. According to the calculation results, the increase of oxygen vacancy which confirmed by XPS measurements will enhance the DOSs of Ce4f orbital, and therefore superexchange interaction of Ce–O–Ce generates magnetism. This work demonstrates an effective route to produce diluted magnetism for CeO_2_ nanocubes and offers a better understanding of room-temperature magnetism in CeO_2_ nanocrystals.
